# Vascular dysfunctions following spinal cord injury

**Published:** 2010-08-25

**Authors:** C Popa, F Popa, VT Grigorean, G Onose, AM Sandu, M Popescu, G Burnei, V Strambu, C Sinescu

**Affiliations:** * >‘Carol Davila’ University of Medicine and Pharmacy, Bucharest Romania; ** National Institute of Neurology and Neurovascular Disorders, BucharestRomania; *** ‘St. Pantelimon’ Clinical Emergency Hospital, Department of General Surgery, BucharestRomania; **** ‘Bagdasar–Arseni’ Clinical Emergency Hospital, Department of General Surgery, BucharestRomania; ***** ‘Bagdasar–Arseni’ Clinical Emergency Hospital, Department of Physical and Rehabilitation Medicine, BucharestRomania; ****** ‘Bagdasar–Arseni’ Clinical Emergency Hospital, Department of Neurosurgery, BucharestRomania; ******* District Hospital Pitesti, Department of Neurosurgery, PitestiRomania; ******** ‘Marie Sklodowska Curie’ Children' Clinical Emergency Hospital, Department of Pediatric Orthopedic Surgery, Bucharest Romania; ********* ‘Bagdasar–Arseni’ Clinical Emergency Hospital, Department of Cardiology, UMF ‘Carol Davila’, Bucharest Romania

**Keywords:** spinal shock, neurogenic shock, orthostatic hypotension, autonomic dysreflexia, deep vein thrombosis, coronary heart disease

## Abstract

The aim of this article is to analyze the vascular dysfunctions occurring after spinal cord injury (SCI). Vascular dysfunctions are common complications of SCI. Cardiovascular disturbances are the leading causes of morbidity and mortality in both acute and chronic stages of SCI.

Neuroanatomy and physiology of autonomic nervous system, sympathetic and parasympathetic, is reviewed. SCI implies disruption of descendent pathways from central centers to spinal sympathetic neurons, originating in intermediolateral nuclei of T1–L2 cord segments.  Loss of supraspinal control over sympathetic nervous system results in reduced overall sympathetic activity below the level of injury and unopposed parasympathetic outflow through intact vagal nerve.

SCI associates significant vascular dysfunction. Spinal shock occurs during the acute phase following SCI and it is a transitory suspension of function and reflexes below the level of the injury. Neurogenic shock, part of spinal shock, consists of severe arterial hypotension and bradycardia. Autonomic dysreflexia appears during the chronic phase, after spinal shock resolution, and it is a life–threatening syndrome of massive imbalanced reflex sympathetic discharge occurring in patients with SCI above the splanchnic sympathetic outflow (T5–T6). Arterial hypotension with orthostatic hypotension occurs in both acute and chronic phases. The etiology is multifactorial. We described a few factors influencing the orthostatic hypotension occurrence in SCI: sympathetic nervous system dysfunction, low plasma catecholamine levels, rennin–angiotensin–aldosterone activity, peripheral alpha–adrenoceptor hyperresponsiveness, impaired function of baroreceptors, hyponatremia and low plasmatic volume, cardiovascular deconditioning, morphologic changes in sympathetic neurons, plasticity within spinal circuits, and motor deficit leading to loss of skeletal muscle pumping activity. Additional associated cardiovascular concerns in SCI, such as deep vein thrombosis and long–term risk for coronary heart disease and systemic atherosclerosis are also described.

Proper prophylaxis, including non–pharmacologic and pharmacological strategies, diminishes the occurrence of the vascular dysfunction following SCI. Each vascular disturbance requires a specific treatment.

Spinal cord injury (SCI) is one of most important health problems worldwide, and one of the most devastating of all traumatic events, with an annual incidence of 15 to 52.5 cases per million of population. About 80% are young males, aged between 15 and 35 years, and 5% are children.[1–6] Neurological disability is frequently encountered, from all spinal cord injured patients 53% are tetraplegic and 42% are paraplegic.

The aim of this article is to evaluate the vascular dysfunctions following SCI. Vascular dysfunctions are common consequences of SCI, and are issues of interest worldwide. Cardiovascular disturbances are the leading causes of morbidity and mortality in both acute and chronic stages of SCI.[7–9]

Impairment in control of the autonomic nervous system, mostly in patients with cervical and high thoracic SCI, causes arterial hypotension, bradycardia, and autonomic dysreflexia. Besides, additional vascular complications, such as deep vein thrombosis and long–term risk for coronary heart disease and systemic atherosclerosis, may occur. 

## Epidemiology

SCI causes important cardiovascular disturbances, with short and long term consequences.[6]  An adequate treatment of the cardiovascular disturbances pays an important role in the therapeutic management of SCI. By reducing the incidence of cardiovascular pathology, morbidity and mortality rates diminish and the quality of life in patients with SCI improves. Cardiovascular disturbances are the most common underlying cause of death, after neoplasms,  in 21.6% cases, and they are contributing causes of death in 18.9% of the cases in chronic SCI.[8] Cardiovascular dysfunctions contribute to 40.5% of the deaths, when both underlying and contributing causes.[8]  The most common causes of death are cardiac failure, atrial fibrillation, atherosclerosis and coronary heart disease, ventricular tachycardia, rupture of abdominal aneurysm, cerebrovascular disease, cardiac arrest, cardiomyopathy and ill–defined heart disease.[8] 

All the patients with motor complete cervical SCI (ASIA A and B) develop bradycardia, 68% develop arterial hypotension, 35% of the patients require vasopressors, and cardiac arrest occurs in 16% of them.[6,10] From patients with motor incomplete cervical SCI (ASIA C and D), 35–71% develop bradycardia, but few have hypotension and require vasopressors.[6] Very rarely they develop cardiac arrest.[6] Thoracolumbar SCI bradycardia is encountered in 13–35% cases.[6] 

The prevalence of autonomic dysreflexia is 48–90% of SCI above T6.[11–13

Deep vein thrombosis occurs in 47–90% cases.[6] Proximal progression of thrombosis and pulmonary embolism appears in 20–50% of cases.[6] Pulmonary embolism causes 35% of deaths.[6]  

Coronary heart disease and systemic atherosclerosis are common findings in patients with chronic SCI, due to the lack of physical activity, obesity, hiperlipidemia, insulin resistance and diabetes, which are more frequently encountered in this group than in the general population.[6] 

## Neuroanatomy

Insula, medial prefrontal cortex, hypothalamus, and cuneiform nucleus project into cardiovascular nuclei within medulla oblongata.[14–16] Parasympathetic impulses are carried out via the vagus nerve. Preganglionic fibers synapse with postganglionic parasympathetic neurons are near the myocardium. There is no parasympathetic outflow for peripheral vessels. 

The preganglionic sympathetic neurons are located within the lateral horn into the intermediolateral nuclei of T1–L2 cord segments. They exit the spinal cord via the ventral root, and synapse with postganglionic neurons from paravertebral sympathetic chain. Postganglionic sympathetic fibers outflow through peripheral nerves vessels and heart. 

## Pathophysiology

Besides the well–known motor and sensitive deficits, autonomic disturbances are frequently encountered. Autonomic nervous system pays a highly important role in the cardiovascular control. Blood pressure and heart rate are controlled by impulses from the autonomic nervous system: sympathetic and parasympathetic. They act antagonistically, according to various needs. The parasympathetic decreases the heart rate. The sympathetic increases the heart rate, myocardic contractibility, raises peripheral vascular resistance and the arterial blood pressure, by inducing vasoconstriction. The arterial blood pressure control depends on the activity of the supraspinal centers, which send activator impulses, through descendent pathways, to sympathetic spinal preganglionar neurons. Secondary to SCI, descendent pathways are interrupted and spinal circuits become incapable to generate sympathetic activity. Disruption of descendent pathways results in sympathetic hypoactivity and unopposed parasympathetic outflow through the intact vagal nerve. Sympathetic hypoactivity results in low resting blood pressure, loss of regular adaptability of blood pressure, and disturbed reflex control. 

## Vascular dysfunctions following SCI

The spinal shock with the neurogenic shock represent a vascular dysfunction that occurs during the acute phase of SCI. Autonomic dysreflexia, coronary heart disease and systemic atherosclerosis are encountered during the chronic phase of SCI. Arterial hypotension and deep vein thrombosis are found in both acute and chronic periods of time. 

### Vascular dysfunctions during the acute phase of SCI

The initial response to SCI is a massive sympathetic stimulation and reflex parasympathetic activity that usually lasts for 3 to 4 minutes and it is mediated by alpha–adrenergic receptors. The hemodynamic effects are severe hypertension and reflex bradycardia or tachyarrhythmia. The massive sympathetic stimulation is due to norepinephrine release from the suprarenal glands. There is also a pressor response caused by the disruption of the cervical and high thoracic vasoactive neurons.[17]

Following this first phase, a massive decrease of sympathetic activity occurs, due to the disruption of descendent sympathetic tracts. The result is physiologically similar to spinal anesthesia and leads to cutaneous vasodilatation, venodilation and reduced venous return, low blood pressure, low cardiac output, bradycardia, bradyarrhythmias and atrioventricular nodal block, due to the lack of vasoconstrictor and inotrope sympathetic activity. The patient is prone to hypothermia, arterial hypotension and bradycardia, by the absence of sympathetic tone and unopposed vagal tone. 

Following this brief hypertensive peak, spinal shock occurs. 

#### Spinal shock

The spinal shock is a physiological disruption of the spinal cord functioning that accompanies SCI. It is a transitory suspension of function and reflexes below the level of the injury. Inputs from autonomic central centers modulate the activity of sympathetic spinal neurons. Sudden disruption of communication between these centers and the sympathetic neurons in the intermediolateral thoracic and lumbar spinal cord leads to spinal shock.

Spinal shock was described for the first time by Whytt in 1750, and the term ‘spinal shock’ was later introduced in the literature by Hall in 1841.[18] The spinal shock's characteristics are sensory deficits, flaccid paralysis, absence of deep tendon reflexes, abolishment of reflex somatic activity, and thermoregulatory disturbances below the level of injury. The spinal shock etiology and pathogenesis remain controversial. Nowadays, there are different theories that partially explain the phenomenon.[18, 19] Spinal shock involves different aspects according to the site of the cord injury. In high cervical SCI, some of the following occur: acute respiratory failure, tetraplegia, anesthesia, lack of all reflexes below injury site, neurogenic shock, detrusor and rectum areflexia, ipsilateral Horner syndrome (ptosis, enophthalmos, miosis, anhidrosis). In low cervical SCI, there is no respiratory failure, because the respiratory muscles are not affected. Patients with high thoracic SCI develop paraparesis. In low thoracic injuries there is no arterial hypotension and no neurogenic shock.[20]  

Neurogenic shock consists of severe arterial hypotension, bradycardia and hypotermia.[20–23]  It is a manifestation of the malfunction of the autonomic nervous system, and it is caused by the absence of sympathetic activity, through loss of supraspinal control and unopposed parasympathetic tone via the intact vagus nerve.[21–24] Systolic blood pressure below 90 mmHg in supine position, which is not the result of low intravascular volume due to hemorrhage or dehydration, is characteristic for neurogenic shock. Severe arterial hypotension usually requires vasopressors.[21,22,25] Severity of arterial hypotension and the need for vasopressors are directly proportional with the severity of the injury.[26] Peripheral vascular resistance and cardiac output deceases, while central venous pressure remains unchanged.[17] 

The spinal shock usually lasts for days–weeks, the average being of 4–12 weeks.[18,20–22,25,27,27–30] There is a lack of consensus on clinical signs, that defines the duration of spinal shock. The appearance of the bulbocavernosus reflex, recovery of deep tendon reflexes or return of reflex detrusor functions are considered by different authors the ending of the spinal shock. Ditunno et al. believe that spinal shock consists of four phases: areflexia or hyporeflexia (0–24 hours), initial reflex return (1–3 days), early hyperreflexia (4 days–1 month) and spasticity (1–12 months).[31]

Neurogenic shock should not be mistaken with hypovolemic shock. In neurogenic shock, hypotension is associated with bradycardia, while in hypovolemic shock tachycardia occurs. In neurogenic shock, the skin is usually warm and dry, except for patients exposed to a cold environment. Neurogenic and hypovolemic shock may coexist, and when this happens, neurogenic shock exacerbates the effects of hypovolemic shock by disabling the vasoconstrictive reflexes that ordinarily preserve blood flow to vital organs. 

### Vascular dysfunctions that occur in both acute and chronic phase of SCI

#### Arterial hypotension

The abolished compensatory vasoconstriction, secondary to changes in sympathetic activity, especially into large vascular beds, like skeletal muscle and splanchnic region vessels, in association with reduced venous blood return, contribute to low blood pressure. Arterial hypotension, especially orthostatic hypotension, improves within days–weeks, thanks to the appearance of compensatory mechanisms involving the vascular bed, skeletal muscles activity, spasticity, increase in the muscular tone, reappearance of spinal sympathetic reflexes, and rennin–angiotensin–aldosterone system.[6] Arterial hypotension is common in both recent injuries, the mean resting blood pressure in tetraplegic patients is of 81 mmHg, and in the chronic tetraplegics, the mean resting blood pressure is of 73 mmHg.[32]

#### Orthostatic hypotension 

Orthostatic hypotension is very difficult to control, and severely impairs the quality of life.[7] Orthostatic hypotension is common following cervical and high thoracic SCI.[7,21,25,33,34] Patients with tetraplegia had a higher prevalence of orthostatic hypotension and a greater fall in blood pressure than patients with paraplegia, irrespective of whether their lesion was complete or incomplete[7,34] It is found in 73.6% of SCI patients, and 58.9% of them have symptoms, such as light–headedness or dizziness.[34] The incidence and severity of orthostatic hypotension diminishes in time. 

The Consensus Committee of the American Autonomic Society and the American Academy of Neurology Orthostatic established in 1996 that hypotension is a decrease in the systolic blood pressure of 20 mmHg or more, or a decrease in the diastolic blood pressure of 10 mmHg or more; this being based upon the assumption of an upright posture from a supine position, regardless of whether symptoms occur.[35]

From clinical point of view, the orthostatic hypotension can be symptomatic, the patients experiencing dizziness, light–headedness, headache, fatigability, nausea, faint, pallor, sweating and loss of consciousness or it can lack any symptoms. 

#### Pathophysiology of orthostatic hypotension

The disruption of efferent pathways from vasomotor center, located within the medulla oblongata, to spinal sympathetic neurons, originating into intermediolateral nuclei of T1–L2 segments involved in vasoconstriction, causes failure of blood pressure regulatory mechanisms and venous pooling.[7,21,33,36] SCI associates significant sympathetic nervous system dysfunctions. This disturbance of sympathetic activity is the consequence of supraspinal control loss over the sympathetic nervous system. SCI with resultant tetraplegia or high paraplegia is associated with significant dysfunction of the sympathetic nervous system. Subsequent complications that occur below the level of SCI are reduced overall sympathetic activity, morphologic changes in sympathetic preganglionic neurons, and peripheral alpha–adrenoceptor hyperresponsiveness. Reduced sympathetic activity below the level of SCI, appears to result in orthostatic hypotension, low resting blood pressure, loss of diurnal fluctuation of blood pressure, reflex bradycardia, and, rarely, cardiac arrest.[26, 37] It is correlated with the severity of SCI. The incomplete SCI, which spare the dorsolateral cord, partially preserving the descending tonic sympathetic supraspinal control to spinal circuits, are less predisposal to low blood pressure.[26] Severe disruption of the descending cardiovascular pathways is correlated with greater abnormalities of cardiovascular control.[26, 38]

The pathological mechanisms responsible for the orthostatic hypotension of SCI patients, still remain unclear. There are a few predisposal factors of occurrence of hypotension in SCI. The etiology of orthostatic hypotension is multifactorial.

## Factors influencing orthostatic hypotension occurrence in SCI:[7]

1. Sympathetic nervous system dysfunction
2. Low plasma catecholamine levels
3. Rennin–angiotensin–aldosterone activity
4. Peripheral alpha–adrenoceptor hyperresponsiveness
5. Impaired function of baroreceptors
6. Hyponatremia and low plasmatic volume
7. Cardiovascular deconditioning
8. Morphologic changes in sympathetic neurons
9. Plasticity within spinal circuits
10. Motor deficit leading to loss of skeletal muscle pumping activity

1. Sympathetic system dysfunction: The sympathetic nervous system plays an important role in cardiovascular control. Sympathetic system dysfunction consists of the disruption of efferent pathways, with supraspinal control loss of spinal centers, both excitatory and inhibitory control with absent or decreased sympathetic tone below the injury level.The parasympathetic outflow is preserved, but the synergy between the parasympathetic and the sympathetic control is lost in patients with cervical or high thoracic SCI. SCI above T6 disrupt the supraspinal control and the sympathetic tone to the splanchnic vascular bed, predisposing the patient to orthostatic hypotension. In order to maintain the systemic blood pressure and the cerebral perfusion in orthostatism, systemic vasoconstriction is induced via an increase in the tonic sympathetic activity and the baroreceptor–mediated mechanisms. The disruption of efferent pathways from the brainstem cardiovascular center to the spinal sympathetic centers causes failure of short–term blood pressure regulation.[7, 21, 23]
  It depends on injury level. It is more severe in cervical or high thoracic, complete SCI. The dynamic test performed on SCI patients and normal individuals stresses the importance of the sympathetic nervous system dysfunction.Both the resting systolic and diastolic arterial pressures are lower in SCI patients than in normal individuals. Following the assumption of an upright seated position the SCI subject has a marked, progressive decrease in systolic and diastolic arterial pressure in the upright posture, the characteristic of the orthostatic hypotension, in contrast with normal individuals who have an increase in the systolic and diastolic arterial pressures.[7]The sympathetic vascular tone, assessed by the Mayer wave power spectrum of systolic blood pressure variability, increases significantly in response to orthostatic posture in patients with injuries below T4, but remains unchanged or decreases in patients with higher–level injury.[39] The systolic blood pressure significantly falls with postural shift in high–level injury group, and does not change in the low–level injury group.[39] The low frequency component in the systolic blood pressure variability is lost in tetraplegic patients, comparative with normal individuals.[40] Cervical spinal sympathetic pathways are instrumental in the genesis of the Mayer waves. SCI interrupt spinal pathways linking supraspinal cardiovascular centers with the peripheral sympathetic outflow and lesions above T3 or more eliminate the normal neural cardiovascular responses to mild orthostatic stress.[39–42]
2. Low catecholamine levels: Plasmatic catecholamine levels, both adrenaline and noradrenaline, are low in SCI patients, comparative to normal individuals. Plasma catecholamines, noradrenaline and adrenaline levels,  are abnormally low in the supine position in both the recent and chronic SCI.[7,25,32,43–46] They do not rise, the way they do in normal individuals, in response to an orthostatic posture or head–upright tilting.[7,25,43–46] This is particularly pronounced in high–level, cervical and high thoracic, complete SCI.[7] This is a marker of failure of sympathetic activity in response to orthostatism or head–upright tilting in the tetraplegic patients, probably caused by the interruption of pathways by which the brain normally controls the sympathetic outflow.[46] In the recently injured tetraplegics, the bladder stimulation caused minimal changes in plasma noradrenaline and adrenaline levels.[32] In the chronic tetraplegics similar stimulation caused marked hypertension, bradycardia and elevation in plasma noradrenaline but not adrenaline levels.[32, 47]
  The elevation in plasma noradrenaline in chronic SCI is caused by a reflex sympathetic discharge from the isolated spinal cord. This response is free of inhibitory impulses from supraspinal center and baroreceptor reflexes, either of which might restrain the increase in blood pressure.[47]
3. Rennin–angiotensin–aldosteron system: The patients with tetraplegia rely on the renin–angiotensin system for orthostatic blood pressure control. In patients with SCI, the plasmatic rennin activity is higher than normal. Plus, rennin levels rise rapidly and much higher in orthostatic posture or head–up tilt, than in normal individuals.[7,46] Rennin release during head–up tilt occur independently of sympathetic nervous activity, and depends on the activation of renal baroreceptors.[44, 46] Due to the elevation in plasma rennin activity, there is a late rise in plasma aldosterone.[46]
4. Peripheral alpha–adrenoceptor hyperresponsiveness: As a compensatory mechanism for the decreased levels of cathecolamines, a peripheral alpha–adrenoceptor hyperresponsiveness below the injury site appears.[48] The hyperresponsiveness may be a consequence of alpha–adrenoceptor hypersensitivity or a failure of presynaptic reuptake of norepinephine at the receptor level.[37]. Peripheral hypersensitivity of alpha–adrenoceptors is responsible for the excessive pressor response during autonomic dysreflexia in patients with high spinal cord injury, and may also contribute to decreased blood flow in the peripheral microcirculation, potentially increasing susceptibility to pressure sores.[37,48,49]In tetraplegic patients following a recent SCI, intravenous infusion of norepinephine  resulted in the elevation in blood pressure higher than normal in normal individuals, probably because of peripheral alpha–adrenoceptor hyperresponsiveness.[32]
5. Impaired function of baroreceptors:Baroreceptors located in the aortic arch, carotid sinus and coronary arteries are involved in the maintenance of blood pressure homeostasis. Baroreceptors detect changes in the arterial pressure and reflex modulate sympathetic and parasympathetic outflow. The impairment in baroreceptor reflex control is encountered particularly in patients with high–level, cervical and high thoracic, injuries, and causes low resting supine blood pressure in SCI individuals.[25]In addition, the circadian rhythms of blood pressure control, including the circulating plasma noradrenaline and cortisol, are abolished in individuals with tetraplegia, and thus high–level lesions.[7, 50] This further highlights the baroreflex dysfunction in high–level SCI. Baroreceptor sensitivity, assessed by using spectral analysis of variability in the heart rate period, RR interval on electrocardiograph, and systolic blood pressure, during orthostatic posture is abnormal in SCI patients with lesions above T3.[39] Baroreceptor sensitivity increases with postural shift in SCI above T3, and it decreases insignificantly in SCI below T3.[39] This mechanism contributes to the high–level injury orthostatic hypotension, above T3. Besides, tetraplegic patients have impaired baroreceptor responses to discrete stimulation of the carotid sinus by using neck suction or neck pressure, whereby both the baroreceptor sensitivity and the range of operation were reduced.[51]Patients sitting in wheel chairs have a chronic loss of stimulation of carotid sinus baroreceptors by routine standing posture. This is associated with diminished sensitivity and reduced buffer capacity of the arterial baroreflex and hypotension.51 Some authors believe that impairment in baroreceptor control is not found exclusively in patients harboring high–level lesions. By using spectral analyses, they demonstrate that paraplegic patients are also prone to orthostatic hypotension. Paraplegic patients also have altered baroreceptor responses to orthostasism and head–upright tilting, whereby the sympathetic response is blunted, and the response of vagal withdrawal was enhanced.[52] So, the impairment of the baroreceptor control, encountered in both high– and low–level lesions, contributes to orthostatic intolerance in SCI.
6. Hyponatremia and low plasmatic volume: The plasma volume is directly dependent on the orthostatic tolerance and postural blood pressure homeostasis.[7] In both acute and chronic SCI, hyponatremia is frequently found.[7,53] The etiology of hyponatremia is multifactorial. Patients have an increased ADH secretion that causes high sodium excretion, decreased renal water excretion, due to both the intrarenal and the arginine vasopressin dependent mechanisms. All these, coupled with habitually increased fluid intake, and the ingestion of a low salt diet and general factors such as the use of diuretics and the intravenous infusion of hypotonic fluids, lead to secondary hyponatremia and low plasma volume.[7,53]
7. Cardiovascular deconditioning appears as a consequence of prolonged bed rest and it is a loss of orthostatic tolerance thought to be related to diminished blood volume, decreased muscle or tissue pressure in the extremities or to functional alterations in the sympathetic nervous system.[54] The cardiovascular decondition is associated with the altered nitric oxide metabolism.[7,55,56]  The altered nitric oxide metabolism causes peripheral vasodilatation, reduces blood pressure, and cerebral vasoconstriction. Increased release of nitric oxide and upregulation of nitric oxide synthase is associated with orthostatic intolerance after prolonged bed rest.[56] Cardiovascular deconditioning is encountered in the acute phase, and its incidence is diminished with the progression to the chronic phase, due to the mobilization of the patient.
8. Morphologic changes into sympathetic neurons:Following SCI, atrophy of sympathetic preganglionic neurons occurs, possibly due to partial deafferentation from the loss of descending medullary input and subsequently axonal sprouting.
9. Plasticity within spinal circuits: Atrophy of sympathetic preganglionic neurons, axonal sprouting and synaptic reorganization may result in possible formation of new and often inappropriate synaptic connections.[17] Spinal reflexes and veno–arteriolar reflexes contribute to the improvement of the arterial hypotension in time.[25]In complete chronic SCI, as a response to noxious stimuli, like a cold pressor test, consisting of the immersion of the foot in ice water for 3 minutes, a reflex sympathetic discharged from the isolated spinal cord results in a significant rise in arterial blood pressure, higher than that in healthy subjects. This response is free of inhibitory impulses from supraspinal center and baroreceptor reflexes, either of which might restrain the increase in blood pressure.[47] There is not enough data to prove that the deafferented spinal cord could generate significant basal sympathetic activity.[17]
10. Motor deficit leading to loss of skeletal muscle pumping activity: A possible mechanism producing orthostatic hypotension in SCI is the failure of skeletal muscle pumps in paralyzed limbs during postural challenge.[7] The absence of skeletal muscle contractions during postural challenge has a powerful effect upon the orthostatic changes in blood pressure.[7]In normal individuals in standing position, postural muscles contractions compress veins and pump blood back to the heart. This is an important way of maintaining venous return in orthostatism. Motor deficit leads to lack of skeletal muscle pumping effect. That would predispose the patient to circulatory hypokinesis by reducing the venous return in the upright position and thus lowering the stroke volume, cardiac output and blood pressure.[7,57,58]The electrical stimulation of the skeletal leg muscles in paraplegic and tetraplegic patients induces muscular contractions, simulating the skeletal muscle pumping effect normally present in healthy individuals while standing. Electrically stimulated leg muscle contractions increased peripheral resistance, and blunted the fall in stroke volume that normally occurs during orthostasis.[7] The significant deceasing in the systolic blood pressure, the diastolic blood pressure and the mean arterial pressure are noted in SCI patients. Functional electric stimulation during the changing position from sitting to standing prevents orthostatic hypotension in SCI patients.[58,59] During the standing position it had equal or even greater effect on improving the blood circulation when compared with voluntary activation in healthy individuals.[58,59] The use of functional electric stimulation during standing and tilting in SCI patients may prevent orthostatic hypotension and circulatory hypokinesis and improve tolerance to tilting and standing.[58,59]

## Deep vein thrombosis 

The risk of developing deep vein thrombosis is highest in 7 to 10 days after the injury and during the early phases of recovery and rehabilitation. The risk diminishes in 8–12 weeks.[6] 

Blood flux diminishes with 50–67% in inferior limbs following SCI, due to the loss of the autonomic nervous system control and to the reduction of the local blood flow.[6]

Factors contributing to deep venous thrombosis occurrence are venous stasis in inferior limbs, secondary to muscle paralysis and lack of muscle pump activity, venodilation, pressure on the calves and hypercoagulability secondary to the reduction of fibrinolitic activity and rising activity of factor 8. Changes in the normal neurologic control of the blood vessels can result in stasis or sludging of the blood.

Signs and symptoms of deep vein thrombosis are leg swelling, vein dilation, increased skin temperature, pain, tenderness, and, rarely, a bluish discoloration of the lower leg. Deep vein thrombosis can be present without any signs or symptoms. 

Thigh deep vein thromboses are a great concern, because the thrombi can dislodge and pass through the vascular tree to the lungs, causing pulmonary embolism, a potentially severe complication of SCI. The major pulmonary embolism has a high mortality rate. There are also no characteristic signs or symptoms of lung clots, signs and symptoms are nonspecific, such as fever, chest pain, cough, or changes in heart rate.

## Vascular dysfunctions during the chronic phase of SCI

The resolution of the acute phase merges with the appearance of hyperactive spinal reflexes, and spastic motor paralysis below the level of injury.[20] The chronic phase of SCI is characterized by motor and sensory deficit, spasticity hyperactive deep tendon reflexes, plantar cutaneous reflex in extension and impairment of gastrointestinal and genitourinary tracts.[17]

### Autonomic dysreflexia

Autonomic dysreflexia, first observed by Anthony Bowlby in 1890, and described by Guttmann and Whitteridge in 1947[11], is the consequence of the interruption of control of the sympathetic spinal cord centers by central nervous centers, and it is a syndrome of massive imbalanced reflex sympathetic discharge to stimuli acting below the level of injury. Distension, stimulation or manipulation of bladder or bowel are frequently determinant factors.[11,27,30,60]

 Autonomic dysreflexia is frequently encountered in cervical injuries. The prevalence of this phenomenon varies between 48 and 90% of SCI above T6.11–13 Some incidence has been reported in SCI as low as T10.[11] Morbidity is associated with hypertension, which can cause retinal, subarachnoid or intracerebral hemorrhage, myocardial infarction, pulmonary edema or seizures. Mortality is rare.[11] Autonomic dysreflexia is characteristic for the chronic phase, but it may occur any time following SCI. In 5.2% cases, it appears before, within the first month from SCI.[7,61,62] 

After the spinal shock resolution, the deafferented spinal cord, in SCI above T6, may generate aberrant impulses to noxious stimuli, leading to autonomic dysreflexia, a life–threatening complication of SCI. Autonomic dysreflexia is a syndrome of reflex sympathetic discharge from the preganglionic neurons in the thoracolumbar spinal cord, occurring in patients with SCI located above the splanchnic sympathetic outflow T5–T6, often triggered by the distension of pelvic viscera. Autonomic dysreflexia is a consequence of supraspinal control loss of sympathetic spinal cord neurons and altered glutamatergic neurotransmission within the spinal cord.[17] The viscerosensitive impulses, below the level of injury, are transmitted through intact peripheral sensory nerves, through ascendant pathways to neurons located within the intermediolateral thoracolumbar nuclei, releasing a sympathetic reflex. Sympathetic hyperstimulation discharge high quantities of norepinephrine, dopamin– beta–hydroxilasis and dopamine, which lead to massive vasoconstriction within arterial system, arterial hypertension, and cerebral vasodilatation. The brain perceives the hypertensive crisis throughout cervical baroreceptors and 9 and 10 nerves. It generates inhibitory impulses that cannot be transmitted below the level of injury. Vasomotor centers from the medulla oblongata try to lower the arterial blood pressure, by parasympathetic stimulation of the heart, through X nerve, generating severe bradycardia.

The development of autonomic dysreflexia is correlated with aberrant sprouting of peptidergic afferent fibers into the spinal cord below the injury. Sprouting of nerve growth factor–responsive afferent fibers has been shown to have a major influence on dysreflexia, perhaps by amplifying the activation of the disinhibited sympathetic neurons.[63]

Peripheral alpha–adrenoreceptor hyperresponsiveness following SCI may play a significant role in the development of autonomic dysreflexia.[7]

There is a constellation of signs and symptoms in SCI above T5–T6 in response to noxious or nonnoxious stimuli below the injury level, including severe arterial hypertension, headache and visual impairment due to cerebral vasodilatation, cutaneous pallor below the injury site, piloerection, secondary to sympathetic activity, reflex bradycardia, profuse sweating and cutaneous vasodilatation above the level of lesion, secondary to parasympathetic activity. Arterial blood pressure can reach up to 300 mmHg, leading to retinal, intracerebral, or subarachnoid hemorrhage, pulmonary edema, myocardial infarction, seizures, confusion and death.[30,64] 

According to the increased blood pressure, there are three severity grades:

1. mild/partial: blood pressure increases < 40 mmHg
2. moderate: blood pressure increases > 40 mmHg, but systolic blood pressure is < 180 mmHg
3. severe: systolic blood pressure increases > 180 mmHg

Coronary heart disease and systemic atherosclerosis: Abnormal lipid profile, with an increase in total cholesterol and low–density lipoprotein (LDL–cholesterol) levels and diminishing of the high–density lipoprotein (HDL–cholesterol) level occur in chronic SCI and lead to a high risk of developing coronary heart disease and systemic atherosclerosis. The reason for diminishing the HDL–cholesterol is still on debate. It is assumed that it is due to an inappropriate diet, adrenergic dysfunction and lack of physical activity.[6] The ratio of total cholesterol/HDL–cholesterol >5 is considered of high risk for coronary heart disease.[6] Goals for optimal cholesterol management currently include an LDL–cholesterol level of < 100 mg/dl, and total cholesterol of < 200 mg/dl.[6] 

## Treatment

### Treatment of neurogenic shock

The treatment of neurogenic shock implies the correction of the arterial hypotension and bradycardia. Treatment of neurogenic shock is individualized for each patient, depending on the severity of the blood pressure and heart rate dysfunctions.  The first two venous lines should be established for the administration of resuscitation fluids and medications.Arterial hypotension, associated with a normal heart rate requires volume loading, with crystalloids and colloids in the first 24–48 hours following SCI. In the absence of concomitant hypovolemic shock, Hartmann's solution implies the administration of 50–100 ml per hour, in order to maintain a systolic blood pressure of > 80 mmHg. Neurogenic shock associated with hypovolemic shock is treated with normal saline or Hartmann's solution in order to increase the intravascular volume and blood pressure. Patients having arterial hypotension with arterial systolic blood pressure < 90 mmHg, and bradycardia with heart rate < 60/minute, require 0.5 mg atropine for low heart rate and sympathomimetics for hypotension. Ephedrine is a sympathomimetic drug used for the treatment of hypotension, and it can be given intravenously 5 mg or subcutaneously 25–50 mg, every 4–6 hours. The heart rate of < 50/minute, nodal or ventricular ‘escape’ dysarhythmias require higher doses of atropine administered as often as necessary up to 2 mg per hour. In severe neurogenic shock, the monitorization of the central venous pressure is mandatory in order to ensure the fluid load up to a central venous pressure of 7–10 mmH_2_O. If the hemodynamic instability persists, a Swan Ganz catheter is assembled. It provides accurate information on cardiac output, heart preload and systemic vascular resistance. In cases of profound hemodynamic instability with low central venous pressure, large doses of vasopressors, like norepinephrine, dobutamine and dopamine are required. Intractable hypotension should raise suspicion of a concealed internal hemorrhage. Tissue perfusion must be maintained. Hypotension, consequence of neurogenic and/or hypovolemic shock causes spinal cord ischemia, cord damage and extent of neurological deficit. In tetraplegic patients blood pressure must be maintained ranging from 80/40 mmHg to 100/60 mmHg. Attention should be paid to fluid overload that can cause cord edema, which will further reduce the tissue perfusion. 

### Treatment of orthostatic hypotension

Nonpharmacological management of orthostatic hypotension: Nonpharmacologic measures should be the first line of therapy.[65] Patients should be advised to avoid precipitating factors of orthostatic hypotension.[65–67] Patients must maintain the plasma volume by increasing the salt and fluid intake.[65, 66] Diuretics, like alcohol and caffeine must be avoided, as well as different vasodilator stresses such as heat stress or common vasodilators, like alcohol. Regular small meals minimize postprandial hypotension.[7,65,67] Compression bandages and/or support stockings are useful in restricting venous pooling in the splanchnic region and limbs.[7,67] The maintenance of the head position  elevated with 10–20 degrees, during the night, increases plasma volume and orthostatic tolerance.[68] Patients with SCI must be told about the symptoms of orthostatic hypotension and they should be encouraged to assume a recumbent or semi recumbent position if they occur.[65]Pharmacological management of orthostatic hypotension: The pharmacological therapy involves the expansion of plasma volume with fludrocortisone [69] or increases the peripheral vasoconstriction with the alpha–adrenergic agonist midodrine.[70,71] Desmopressin acetate and erythropoietin are useful supplementary agents in patients with more refractory symptoms.There are rare patients who will require additional agents to treat their symptoms.[7] Nitric oxide synthase inhibitors are under investigation, and the result seems promising. They normalize the supine blood pressure in SCI with tetraplegia.[56]Associated pathology, such as type 2 diabetes mellitus, progressive renal and cardiovascular disease often requires additional treatment. Angiotensin–converting enzyme inhibitors are often necessary for progressive renal and cardiovascular disease. Tetraplegic patients are tolerant of an acute bout of orthostatic stress after partial angiotensin–converting enzyme inhibitors administration. Although the mean arterial blood pressure, is reduced immediately after angiotensin–converting enzyme inhibitors administration, it is maintained after that, with increased angles of tilt and no symptoms occurrence.[72]

Treatment of deep vein thrombosis:Prophylaxis of deep vein thrombosis is mandatory. Nonpharmacologic prophylactic strategies include the mobilization of the patient, thigh high compression stockings, pneumatic calf compression boots and physical therapy. Pharmacologic prophylactic treatment consists of anticoagulant therapy, such as heparin, low molecular weight heparin or oral anticoagulants.Heparin administered subcutaneously is useful in reducing blood viscosity and improving flow. Low molecular weight heparin has been demonstrated to be superior to standard heparin preparations and the former also significantly reduces the incidence of bleeding. Because of the high risk of developing deep vein thrombosis, in patients with SCI, regular measurement of bilateral calf and thigh circumferences is mandatory. Doppler ultrasound or venograms are frequently performed to establish baselines. If deep vein thrombosis occurs, intravenous heparin is administrated for 7 to 10 days. Once adequate anticoagulation is provided, the patient is switched to an oral anticoagulant therapy for 3 months. In patients in whom thromboembolism occurs under anticoagulation therapy or for patients with high risk to anticoagulant therapy, an inferior vena cava filter can be placed.

Treatment of autonomic dysreflexia: The avoidance of trigger factors prevents the appearance of this phenomenon. Sympatholitic drugs administration (alpha–adrenergic blocking agents, ganglionic blockers, catecholamine depleters), vasodilator drugs and local anesthetics can prevent the onset of this phenomenon.[12,73,74] Oral terazosin is administrated prophylactically. The treatment of autonomic dysreflexia consists of an elevated position of the head and trunk and sublingual administration of nifedipine or nitroglycerine for an immediate effect. Other drugs used are mecamylamine, diazoxide, and phenoxybenzamine.[11] The main therapeutic issue is the removal of the trigger factors, bladder and bowel decompression, because the persistence of the visceral stimuli maintain the sympathetic response.[30] The analgesia is obtained by the administration of paracetamol or co–proxamol, aspirin or non–steroidal anti–inflammatory drugs being contraindicated.[27] Unresponsive patients or the ones with poor response to therapy are suitable for regional or general anesthesia, that successfully blocks sympathetic response.[13] Treatment of dyslipidemia: Lipid–lowering drugs are used for dyslipidemia. 
